# NICU nurses’ ambivalent attitudes in skin-to-skin care practice

**DOI:** 10.3402/qhw.v9.23297

**Published:** 2014-02-20

**Authors:** Ingjerd G. Kymre

**Affiliations:** Center for Practical Knowledge and Institute for Nursing and Health, PHS, University of Nordland/UiN, Bodø, Norway

**Keywords:** Reflective lifeworld research, SSC, Kangaroo Mother Care, NICU nursing, parent–infant separation, phenomenology, developmental care

## Abstract

This article illuminates the essence of Neonatal Intensive Care Unit (NICU) nurses’ attitudes in skin-to-skin care (SSC) practice for preterm infants and their parents. Health care providers are in a unique position to influence the dynamic between infants and parents, and SSC affects both partners in the dyad. The design is descriptively phenomenological in terms of reflective lifeworld approach. Eighteen Swedish, Danish, and Norwegian nurses from NICUs offering varied possibilities and extents of SSC participated. NICU nurses’ attitudes in SSC practice are ambivalent. The nurses consider the sensory, wellness, and mutuality experiences to be primary and vital and enact SSC as much as possible. But “as much as possible” is a broad and varied concept, and their attitudes are ambivalent in terms of not always facilitating what they consider to be the optimal caring conditions. The source of NICU nurses’ ambivalent attitudes in SSC practice is a complex interplay of beliefs, norms, and evidence, which have a multidisciplinary basis. The ambivalent attitudes are, to a great extent, the result of the need to balance these multidisciplinary concerns. This needs to be acknowledged in considering SSC practice, as well as acknowledging that clinical judgments concerning optimal SSC depend on parents and infants unlimited access to each other, which NICU nurses can influence.

Skin-to-skin care (SSC) in terms of facilitating and enacting situations of direct skin-to-skin contact between preterm infants and parents is common in Neonatal Intensive Care Unit (NICU) nursing practices. Human physical contact is a powerful contributor to health and well-being, and it is in the arms of their caregiver that the infant begins developing the vital capacity for human connection and meaning (Duhn, 2010). SSC is well known as a main component of the Kangaroo Mother Care (KMC) method. KMC is defined as “early prolonged and continuous skin-to-skin contact between a mother and her newborn low-birth-weight infant (<2500 g. viz. preterm and/or low birth weight infant) both in hospital and after early discharge, with (ideally) exclusive breastfeeding, and proper follow-up” (Nyqvist et al., 2010). Research on KMC suggests that it involves at least the following positive outcomes: provides thermal regulation, enhances bonding, helps reduce maternal postpartum depression symptoms, increases parental sensitivity to infant cues, helps establish breastfeeding and increased duration of exclusive or any breastfeeding, and positively affects infant–parent psychological development and the development of mutual communication (Nyqvist et al., 2010). According to Charpak and Ruiz (2011), the kangaroo position was initially regarded as a modality that was not necessary or feasible in high-tech settings. However, the expert group and the 7th KMC international workshop recommend enhanced practice of KMC, including continuous skin-to-skin contact whenever possible in high-tech NICUs (Nyqvist et al., 2010).

Health care providers are in a unique role to influence the dynamic between infants and parents (Duhn, 2010). SSC affects both partners in the dyad and reduces physiological stress in mothers in the first postpartum weeks (Bigelow, Power, McLellan-Peters, Alex, & McDonald, 2012). Skin-to-skin contact promotes physiological stability and interactions between parents and preterm infants, and is considered to be a developmental care practice that may have an important impact on brain maturation when administered over an extended period (Scher et al., 2009). As an example, SSC improves sleep, and neurological development depends on sleep (Ludington-Hoe, 2011b). In other words, SSC has increasingly been seen as part of the treatment arsenal of the NICU.

The close proximity of mother and infant in SSC facilitates the mother's ability to recognize and become familiar with the infants signals (Bigelow, Littlejohn, Bergman, & McDonald, 2010). Although the ideas of parental involvement in the care of babies and use of Kangaroo Care (KC) are widely accepted in NICUs, their translation into specific policies and practices varies, both within and between countries (Pallás-Alonso et al., 2012). In asking why KMC is regarded as a standard for optimal quality of care in some well-respected care settings and looked at as “complementary and alternative” medicine in others, it is suggested that both insufficient access to appropriate scientific information and resistance to changing time-honored practices are at the root of these discrepancies (Charpak & Ruiz, 2011). The dosage of the care has varied widely from less than 1 to 24 h a day, but at least 1 h is recommended to complete one full cycle of sleep according to a review article by Ludington-Hoe (2011b). Neonates in Sweden, Denmark, and Norway receive an average of 3–6 h a day skin-to-skin, which means their remaining hours are spent in less optimal environments (Olsson et al., 2012).

Previous research has identified that NICU nurses’ attitudes to SSC are positive and supportive, however practical concerns were addressed about safety in low-birth-weight infants, organizational support, noise, and the need for clear protocols, especially for low-birth-weight infants (Chia, Sellick, & Gan, 2006). Barriers to the use of KC have been centered on issues of infant safety, maternal readiness, and institutional support (Ludington-Hoe, Morgan, & Abouelfettoh, 2008). In Nordic NICUs, staff members consider the lack of space in the intensive care room an important barrier to SSC (Olsson et al., 2012).

Available accommodations and a family-friendly environment influence parental presence and motivation to be with their child in the NICU (Heinemann, Hellström-Westas, & Nyqvist, 2013; Wigert, Berg, & Hellstrøm, 2010).

This article is part of a larger study on SSC from the perspective of NICU nurses. Its contribution to the body of research on SSC is to emphasize NICU nurses’ lived experiences as providers who implement and influence SSC, by describing the essence of their attitudes in the practice. The main term SSC will be used interchangeably with KMC and KC according to how they are used by participants and references.

## Aim

The aim of this article is to illuminate the essence of NICU nurses’ attitudes in SSC practice for preterm infants and their parents.

## Method

The approach of reflective lifeworld research as developed by Dahlberg, Dahlberg, and Nyström (2008) was chosen, which in turn is based on the phenomenological philosophy of Husserl, Heidegger, Merleau-Ponty and Gadamer (Dahlberg et al., 2008, p. 24). It is an empirical application that is outlined by drawing on their philosophy of science, which makes the sphere of lived experience the starting point of all meaning. This approach attempts to grasp the meaning of phenomena as they are given in lived experience. In the present article, the analysis can be described as descriptively phenomenological. The approach assumes an open attitude to the phenomenon, in this case the essence and its constituents of how NICU nurses’ attitudes to SSC manifest themselves in their lived experience of practicing it, or in other words, how they enact and experience it. The open attitude is described in terms of “bridling,” which allows the researchers to see what is well-known in a new light (Dahlberg et al., 2008, p. 121). The approach involves alertness, and a willingness to listen, see, and understand, to allow the deeper meaning of phenomena to come to expression. Hereby we understand phenomenology as a qualitative science of phenomena, which tries to analyze and articulate them scientifically, which is to say critically and thoroughly. Meaning means the intentional meanings that are being born in the relationship between subject and phenomenon, and bridling means to reflect upon the whole event when meaning comes into being, according to Dahlberg et al. (2008, p. 132). Meanings are often implicit, tacit, and taken for granted in the natural attitude (2008, p. 36), and it is directly related to the understanding of phenomena (2008, p. 48). The aim, in line with Dahlberg et al. (2008, p. 115), is to describe, clarify, and elucidate the lived world in a way that expands our understanding of human being and human experience, and the clarification of meaning as it is given through the nurses’ descriptions from the context of SSC practice in NICUs. In carrying out phenomenological analysis the goal is to describe the phenomenon's essence, its essential meanings, and the structure of meanings, according to Dahlberg et al. (2008, p. 245).

### Participants

A purposive sample of 18 nurses from three NICUs in Sweden (S), Norway (N), and Denmark (D), six from each, were interviewed at their workplace. Local unit leaders had been asked to transmit a written inquiry to the staff, and they were helpful in selecting the participants from the criteria that they were willing to participate, and available to be interviewed during two specific days and afternoons. Nurses with NICU practice for more than 5 years were prioritized to ensure practical experience. The nurses had practiced S: 3–24 years (median 13), N: 4–22 years (median 11), and D: 7–22 years (median 12) in an NICU. Twelve had a higher degree of pediatric, neonatal, intensive, surgery or public health nursing, NIDCAP-education (Newborn Individualized Developmental Care and Assessment Program), or other specialized courses. Two nurses, who had practiced less than five years due to maternity leave, were included. All were female, though this was not a criterion. The sample represents both continuous and intermittent SSC practice; two units represent all preterm newborns, including extremely preterm; and one unit from 28 gestational weeks. The Danish NICU was the oldest and most crowded, and the others were comparatively new. The Swedish unit had beds, the others, recliner chairs available for SSC.

The choice to use informants from three Nordic countries was not meant for comparison, but it was based on the assumption of a common history and culture of NICU care across these countries. However, in addition to significant common ground, the practices in Norway, Sweden, and Denmark would simultaneously provide sufficient variation and nuance to give a more comprehensive treatment of the phenomenon under investigation, as well as a Nordic perspective on meaning within SSC. In the search for essences, the descriptions must be rich in order for essences to be found, and they should include many aspects and nuances, according to Dahlberg et al. (2008, p. 248).

### Interviews

The individual interviews were conducted in quiet rooms separated from the NICU activity. The nurses were prepared, interested in participating, and had read the information letter beforehand or before conducting the interviews. The nurses were asked to openly describe experiences concerning SSC practice, and to tell how they facilitate, enact, experience, and consider it in everyday NICU practice. To explore what the participants considered important, probing questions were asked to obtain details, and to clarify unclear statements. They were also asked about their reflections on the issue that SSC sometimes is called nursing intervention and sometimes medical treatment or technique, with the purpose to open up that area, which is in line with Dahlberg et al. (2008, p. 191), before ending the interviews. All spoke in their own mother tongue, but because all respondents were Scandinavian, mutual understanding was unproblematic. The digitally recorded material was transcribed verbatim by the author during spring 2010. The first author, who carried out the open dialogue interviews alone in November and December 2009, had no connection to the NICUs.

### Ethical considerations

This study was approved by the regional commitee for medical and health research ethics (REK), which carries out an assessment as to whether it was undertaken in an acceptable manner (document reference: 2009/106-18). It is also approved by the data protection official for research, Norwegian Social Science Data Services (NSD project number: 22199). The material was stored according to guidelines of the National Committee for Research Ethics in the Social Sciences and the Humanities (NESH, 2006) and Ethical guidelines for nursing research in the Nordic Countries (2003). Permission to carry out the interviews, based on written information about the study, was obtained from the respective unit leaders in all countries. A letter from the first author introduced the participants to the aim of the interview and the participating criteria. Permission to record the interviews was given by each participant, who was assured that the information would be treated confidentially. They were informed that participation was voluntary and about their right to withdraw from participating before they signed an approval according to NESH (2006) and Dahlberg et al. (2008, p. 202).

### Analysis

A descriptive phenomenological analysis was chosen for this study, which aims at faithful description of the essential meaning structure of the phenomenon. To avoid misconceptions of meaning, the original languages were kept as long as necessary during transcription and analysis, until their essences were found and translated to English. In line with reflective lifeworld research, the entire interview text was initially read to get a sense of its wholeness aspect. The phenomenological analysis of the main material that consisted of naïve descriptions from the NICU nurses of the phenomenon of interest was carried out in the approach of “bridling,” in line with Dahlberg et al. (2008, p. 241). An attitude of reflection was adopted in the scientific sense that smaller meaning units were identified, by dividing the whole into parts with respect to which the meaning was seen. In this case, in following the concept of “bridling,” careful attention was paid to how the phenomena and its meanings were made explicit. Meanings that seemed to belong to each other were temporarily put together in clusters, and then related to each other in looking for essential meanings and structures that describe the phenomenon. This part was conducted by always seeing the parts in terms of the whole, and by being sensitive to nuances and changes in meaning, described as a movement between figure and background by Dahlberg et al. (2008). All clustered meanings were related to each other to form a pattern that describes the phenomenon as that very phenomenon. The presentation of the phenomenon was then described in terms of the essence, followed by an identification of its constituents, which are the meanings that constitute the actual essence. Nuances that were present in the original data will be shown in the constituents.

## Findings

NICU nurses’ attitudes in SSC practice are ambivalent. The nurses consider the sensory, wellness, and mutuality experiences primary and vital, and enact SSC as much as possible. But “as much as possible” is a broad and varied concept, and their attitudes are ambivalent in terms of not always facilitating what they consider to be the optimal caring conditions for preterm newborns and their parents. The attitudinal ambivalence is manifested in the following constituents: Their professional foundation of SSC is ambivalent in terms of whether to consider it nursing care or medical treatment. The assessments of whether to prefer SSC or incubator treatment for unstable infants are varied and indicate a significant dilemma in terms of choosing between security and possible recovery and stabilizing wellness. The NICU nurses’ enactments of prolonged and continuous SSC are diverse and ambivalent in how they express the meaning of doing it “as much as possible.” They experience both being supported and challenged by scientific research evidence on SSC in that their beliefs and experiences are acknowledged, but their established practices were challenged. Scientific evidences and guidelines are increasingly challenging their established practices within which they signal a legitimating of separation between preterm infants and parents on one hand, and promote SSC as developmental care on the other hand. However environmental conditions for parental presence in the NICU are considered the most important obstacle for optimal enactment of SSC because these often do not facilitate its enactment on either the infants or the parent's premises.

### An ambivalent professional foundation of SSC

SSC is considered vital for parents and infants in that it involves “more than the physical contact.” It is discussed in terms of wellness and as leading to mutuality, attachment, growth, and development. But there is ambivalence in whether to designate SSC as the caring practice of nurses or as medical treatment. The term care is preferred, but many are more or less willing to assign SSC the status of medical treatment or technique in order for its vital importance to be acknowledged, which make their attitudes ambivalent.

A nurse said, *“*I appraise SSC as an important part of the treatment and it treats the whole person, thus it is natural and not a medical technique” (S). But there is also resistance to describing SSC as a medical technique or treatment; hence it is considered comprising more than replacing technical equipment, but “it is a double matter in that without SSC, I consider something is missing in the medical treatment” (D). In addition, a nurse said: “To establish SSC practice we need to communicate it as medical treatment, not only as important for attachment purposes, because some will experience that as a bit fuzzy” (S). A more relaxed quotation was: “I do not care what we call it, I can see it works” (N).

Enactment of SSC is something the nurses consider commonplace and is based on their initiative and judgments, such as: “Nurses often decide when the infants are ready for SSC, and the doctors usually support us in that decision” (N). They would rather settle SSC as the first choice. On the one hand, the descriptions indicate a resistance to assigning it to the provenance of doctors. On the other hand, there is a wish that more doctors would be engaged in SSC in terms of legitimating it as a necessary condition for developmental purposes.

Expectations were addressed to nurses as well, such as:

What nurses do is the most important in developing NICU care, I think, because the technological treatment is already at an advanced stage. We need to focus more on what nurses do

(N), she said, in addressing responsibility of nurses in acknowledging and promoting SSC.

Particular examples of practicing SSC such as using it in situations of asphyxia and cooling were exemplified: “In extraordinary instances of concern on behalf of the infant we should use SSC, because that is what they need” (D). Other examples of using SSC because of particular concerns were mentioned, for example newborns of drug abusers or absent parents, and very ill infants. These latter examples indicate a legitimating of SSC as something the infants need for therapeutic or medical purposes. Another ambivalence is evident here inasmuch as SSC is considered both a new treatment resource and at the same time, is spoken of in terms of *getting back to basics* in how they enact SSC, in the sense of humanizing the care. The nurses considered both the terms caring and medical treatment are manifold and that the term medical treatment is on the move.

### Supported and challenged by scientific research evidence

Arguments used to encourage or promote SSC are, according to the descriptions, often drawn from practical experience and from institutional education. Comments indicate that they find well-known effects from SSC cohere with their own practical experience, which strengthens and support their beliefs; “Actually, we have been practicing SSC for a long time, but we do it increasingly intensively, and we have understood its importance, which we tell the parents” (N). Although the participating nurses rarely referred to scientific articles, evidence based knowledge of effects and benefits were mentioned as affirmative to their belief in general. Aims, in terms of “comfort” and “getting to know each other” are often used in encouraging parents to hold their infants skin-to-skin. In perceiving resistance and trying to encourage parents, however, they argue by referring to the most known physiological effects of SSC, such as improved breastfeeding, growth, and brain development.

In general, the nurses described increased use of SSC: “we are more aware, and enact SSC for more hours and more systematically” (D). Their focus on the infants’ cognitive and brain development seemed to be strong. They remarked the challenge that there is always something they expect to improve or to be improved in SSC practice, pointing to expectations from themselves and colleagues, as well as from the institutional level. One nurse reflected: “According to the huge effects we see in the infant, SSC is important and needs to be improved, and increasing research might give it a higher level of status” (S), indicating a belief in science to give the practice legitimacy.

### Varied assessments of whether to prefer SSC or incubator for unstable infants

The nurses were discouraged by varying attitudes to the impact of SSC amongst physicians and nurses when infants are unstable, as exemplified by: “Unstable infants with alarming monitors need SSC, I think, but some physicians think they need to stay in the incubator for the same reason” (D). This issue implies restrictions in transferring infants. Usually, SSC is established from the nurses’ initiative, except for the smallest infants, for whom there are medical restrictions the first week: “Sometimes their skin is immature and there is water loss, and SSC is restricted in agreement with the medical staff” (S). An example of preferring SSC is this; “No newborn infant is too sick for SSC except if they have a thorax drain, then it is too risky. I believe it will help the sickest in many situations, however” (D). Clashes of interests of both enthusiasm and resistance in staff members were expressed, which to some extent makes the enacting of SSC a matter of individual judgment.

Descriptions concerning security for fragile infants indicate the consideration of risk in transfer as an obstacle to SSC activity, as exemplified by this reflection:

If the infant is very ill and unstable and cannot cope with so much manipulation, we have a problem in the act of transferring, which we have to assess within every new instance

(N). The challenge of transferring is doubled in that normal practice has been to transfer unstable infants back to the incubator, but it is increasingly normal to stabilize the infants on the skin. However, ambivalence is shown in that reduced access to the tiniest and most unstable infants can be experienced as challenging the priority of non-separation, because their access to the infant is easier in the incubator. But there is a general understanding of coherence between wellness and medical treatment as is evident from the following:

I suppose we could treat an infant in an incubator and make him grow, but I mean, if you take something that basic as wellness and proximity from the infant it will be more difficult to obtain medical goals, so my point is we have to work with these matters together. (N)

Being physically close to parents bodies sometimes makes nurses nervous in critical situations, but SSC is given priority in that the alternative of transferring the infant to the incubator is considered even more risky. The mood of the parent is sometimes challenging: “Some parents are sharp, critical, or angry, which makes it difficult to work so closely” (S), but working close to parents is unproblematic for others, which makes it an individual concern.

### Diverse enactments of prolonged and continuous SSC

In contrast to a general expression of providing SSC as much as possible, the articulated norm for the extent of SSC varies between and within the units, from 4–6 h/day to 24 h/day. Those norms were expressed both as institutional and individual, and manifest themselves through descriptions of environmental limitations and through how individual nurses enact SSC.

It is a concern, among nurses when newborns are left alone in incubators or beds during the night. A concern for many is the unavailability of caregivers when the infant signals a need to be comforted; vague signals from the infant might not be perceived by anyone.

We do not make parenting easy when we say it is ok if they leave for the night. We are sending mixed signals in encouraging them to stay with the infant and then saying that the infants does not need them for the next 12-13 hours. (D)

One of the nurses said she did not like to think about how many hours the infants were deprived of that contact, and she calls it a dilemma not to be able to have parents stay the night. Another nurse exemplifies the dilemma this way:

If a newborn infant has been home with the mother, she wouldn't dream of leaving him alone at a hospital, but she is there with the child. However if the newborn is in a NICU, there is no bed nearby for her, - it is a strange system, isn't it? (D)

Others provide SSC 4–6 h a day and do not describe it as minimal, which indicates varied attitudes to the idea of non-separation. But another aspect of this problem has to do with the fact that not all parents are equally willing to stay the night. One nurse said: “Some parents arrive late and leave early, and sometimes we find it too hard to tell them the infant needs them, so maybe we should demand they stay” (S), which indicates a moral aspect of collaboration between staff and parents.

Some parents find it hard to manage SSC for more than a few hours a day, or are not willing to do so. Some find it problematic to leave siblings who need them as well. The nurses therefore realize they have to balance how they support parents, all of which exacerbates the dilemma of providing SSC. One statement, “SSC around the clock would be demanding, because we have an intense job, and I cannot imagine a situation like that to be honest” (N), exemplifies a certain resistance to continuous SSC. But although this attitude is common it is also changing. This nurse noticed a generally positive attitude to the practice in the daytime.

The NICU nurses pay attention to some parents’ problems of getting enough sleep in SSC at night, and sought to offer practical assistance such as arranging for parents to sleep nearby. The nurses call attention to limitations for many families, which affects the continuity and extent of SSC; “knowing so much about estimated optimal conditions is a great challenge, thus physical limits force us to adapt everything to the existing conditions for SSC” (D). The limitations are first and foremost related to the possibility for parents and families to relax, eat, and live a family life, with an unlimited access to the infant. In the N and D units, routines are not adjusted for continuous SSC, and they dislike signaling that it is optimal if parents stay and alright if they leave. An ambivalent attitude is revealed when imagining being in the mother's situation:

We aim at SSC, but sometimes I am ambivalent in transferring an infant with a tube from the incubator, because the process is time-consuming and I am responsible if something happens. If the preterm was my baby, I would have considered it cruel not to hold him, however. (N)

This example illustrates the role empathy plays in legitimating SSC for an unstable preterm. This nurse's reflection indicates a kind of legitimating of SSC through imagining being in the parents’ place and at the same time admits that the situation is considered demanding for the nurse, which is a further ambivalence.

A nurse who had participated in a process of evolving SSC in terms of KMC-practice 24 h/day, said:

It is not only about changing attitudes, because it is a new way of working with a complex intertwining in including parents much more in the care. Nothing is negative about it, it is just demanding, especially in the beginning. (S)

## Discussion

NICU nurses’ attitudes in SSC practice are essentially ambivalent. The source of the ambivalent attitudes is complex and nuanced and illuminates the phenomenon's interplay of beliefs, norms, and evidences in SSC practice. The ambivalence typically implies that the NICU nurses do not always do what they consider is right and optimal in SSC practice, yet their belief in a vital importance of this care is obviously strong. Scientific research is increasingly challenging their established practices in which they tend to accept separation between preterm infants and parents. Their attitudes to SSC practice are connected with parents’ possibilities and their premises such as willingness and presence. Because environmental conditions such as lack of accommodations for parents in or near the unit, often hinder the NICU staff in offering prolonged or continuous SSC, they practice care that is not optimal, which makes it an ethical issue. Those environmental conditions have been discussed in previous research as institutional obstacles (Chia et al., 2006; Heinemann et al., 2013).

As illuminated in this study, it is not only a concern whether to offer continuous or intermittent SSC, but is also an issue concerning separation or non-separation in terms of the parents’ access to the infant and the infant's access to the parent to achieve mutuality and comfort. This is in line with Ludington-Hoe (2011a), who claims: “Until nursing professionals endorse and routinely practice the new paradigm of non-separation of infant and mother during the neonatal period, optimal KC practice will not exist.” Flacking, Thomson, Ekenberg, Lövengren, and Wallin (2013), suggest that co-care facilities *per se* do not necessarily increase skin-to-skin contact, but are, together with supportive nursing staff, a practice vital for the implementation and usage of skin-to skin contact, and Heinemann et al. (2013) illuminate the importance of the parents’ unrestricted physical closeness with their infant and suggest there should be parent accommodation in the NICU.

In this study we have seen that nurses’ attitudes to parental presence in the NICU at night are diverse and complex. It is necessary to balance parents need to sleep with the infants need to have access to a parent when signaling a need to be comforted, as well as the recommendations of continuous SSC 24 h/day. This illuminates the complexity of attitudes to SSC and continuous SSC practice. These can be a conflict in that nurses do not always do what they consider optimal. There is a noticeable tension between loyalties to established practice on the one hand, and providing SSC as much as possible on the other hand, which also is a source of the ambivalence. However, new paradigmatic bodies of knowledge such as developmental care and family centered care, appear to challenge established practice which has been the horizon within which the NICU nurses act and reason. Altimier and Phillips (2013) claim ignorance of providing neuroprotective care for preterm infants is no longer acceptable, and say “as the preterm infant matures, the quality of the environment in which the infant resides plays a critical role in the trajectory of recovery, growth, and development.”

In this study, SSC is enacted “as much as possible” in terms of a broad and varied concept of what that means. However, SSC or KMC is recommended by the terms “as much as possible,” “as long sessions as possible or as long as the parents are willing to,” “early initiation and extended use,” “the more the better” and “as much as they wish” (Altimier & Phillips, 2013; Blomqvist, Frölund, Rubertsson, & Nyqvist, 2012; Blomqvist & Nyqvist, 2010; Ludington-Hoe, 2011b; Nyqvist & Heinemann 2011). Because these recommendations are essentially imprecise, there is a clear need for individual judgments as well, in situations as described in this study, for example in assessment of security for unstable infants, or parents’ need to sleep. According to Patricia Benner (2000), clinical judgments cannot be separated from ethical reasoning because each clinical judgment judges what good is at stake and what to do in each particular situation, which depends on an understanding of worthy ends in nursing practice. According to Ludington-Hoe (2011b), the physiological stabilizing abilities in SSC may be life-saving and beneficial. However, the ambivalent attitudes in this study emphasize the need of individual judgments in each particular situation where distinct benefits, wellness, and security need to be balanced or ethically judged both by NICU nurses and physicians.

The growing body of research that promotes SSC as developmental care gives it a multidisciplinary foundation which builds on nursing care, ethics, medicine, social science, and psychology. Aita and Snider (2002) suggested that the concept of developmental care is “predicated on a principle of interprofessional collaboration and represents a step towards the establishment of multidisciplinary care giving, where knowledge is shared in common goals.” In following their suggestion and this study, SSC as the core of developmental care has a multidisciplinary foundation. This means in turn that the ambivalence in the attitudes of nurses is to a great extent the result of the need to balance these multidisciplinary concerns.

### Methodological considerations and limitations

Open interviews have limitations as dialogues, and therefore written information was sent to the informants in order to clarify and limit the study and the phenomenon to be investigated. The time used for the interviews was determined by the researcher, but this was no obstacle to obtaining of the phenomena. In spite of different Scandinavian languages that could be considered a limitation for the interviews and analysis, the level of understanding was fully adequate and all unclear expressions were clarified at once.

## Conclusion

NICU nurses’ attitudes in SSC practice are ambivalent. The nurses consider the sensory, wellness, and mutuality experiences to be primary and vital and enact SSC as much as possible. But “as much as possible” is a broad and varied concept, and their attitudes are ambivalent in terms of not always facilitating what they consider to be the optimal caring conditions. The source of NICU nurse's ambivalent attitudes in SSC practice is a complex interplay of beliefs, norms, and evidence, which have a multidisciplinary basis. The ambivalent attitudes are, to a great extent, the result of the need to balance these multidisciplinary concerns. Clinical implications thus involve acknowledging the need to balance multidisciplinary based knowledge and concerns in considering SSC practice as well as acknowledging that clinical judgments concerning optimal SSC depend on parents and infants having unlimited access to each other, which NICU nurses can influence.
